# A Review of *Pseudomonas aeruginosa* Metallophores: Pyoverdine, Pyochelin and Pseudopaline

**DOI:** 10.3390/biology11121711

**Published:** 2022-11-25

**Authors:** Ghassan Ghssein, Zeinab Ezzeddine

**Affiliations:** Laboratory Sciences Department, Faculty of Public Health, Islamic University of Lebanon (IUL), Khalde P.O. Box 30014, Lebanon

**Keywords:** *Pseudomonas aeruginosa*, metallophores, pathogenesis, pyoverdine, pyochelin, pseudopaline, metal ions

## Abstract

**Simple Summary:**

The human pathogen *Pseudomonas aeruginosa* (*P. aeruginosa*) causes several infections, both acute and chronic, mainly in hosts with compromised immunity and in patients suffering from cystic fibrosis. The pathogenesis of this bacterium is caused by several factors. Metallophores, which are molecules for sequestering metal ions, are among these factors. *P. aeruginosa* secretes three types of metallophores, two for iron chelation (siderophores) named pyoverdine and pyochelin and the third one (pseudopaline) which can chelate zinc, nickel and cobalt. This review sums up in one paper all the important characteristics of these three metallophores, including their biosynthesis process, secretion, uptake after metal chelating and genetic regulation.

**Abstract:**

*P. aeruginosa* is a common Gram-negative bacterium found in nature that causes severe infections in humans. As a result of its natural resistance to antibiotics and the ability of biofilm formation, the infection with this pathogen can be therapeutic challenging. During infection, *P. aeruginosa* produces secondary metabolites such as metallophores that play an important role in their virulence. Metallophores are metal ions chelating molecules secreted by bacteria, thus allowing them to survive in the host under metal scarce conditions. Pyoverdine, pyochelin and pseudopaline are the three metallophores secreted by *P. aeruginosa.* Pyoverdines are the primary siderophores that acquire iron from the surrounding medium. These molecules scavenge and transport iron to the bacterium intracellular compartment. Pyochelin is another siderophore produced by this bacterium, but in lower quantities and its affinity for iron is less than that of pyoverdine. The third metallophore, pseudopaline, is an opine narrow spectrum ion chelator that enables *P. aeruginosa* to uptake zinc in particular but can transport nickel and cobalt as well. This review describes all the aspects related to these three metallophore, including their main features, biosynthesis process, secretion and uptake when loaded by metals, in addition to the genetic regulation responsible for their synthesis and secretion.

## 1. Introduction

The *Pseudomonas* genus comprises more than 120 species of rod shaped, Gram-negative and flagellated bacteria that are predominant in humid environments including water and soil [1,2]. These bacteria can be pathogenic to humans, plants and animals [3]. Gram-negative bacteria have two membranes, a peptidoglycan thin layer encircled by an outer membrane containing lipopolysaccharide (LPS) [4]. The latter is made up of three units: a hydrophilic polysaccharide, O antigen, and lipid A, which is a hydrophobic domain, responsible for the endotoxic activity of these pathogens [5]. *Pseudomonas aeruginosa* (*P. aeruginosa*) strains mostly associated with causing human infection are γ-*Proteobacterium* [6]. *P. aeruginosa* causes serious nosocomial infections, in addition to infections that can be fatal in immunocompromised people and persons suffering from physical damages (e.g., burn wounds) in addition to chronic infections in patients with cystic fibrosis [7,8]. In addition, it causes many other diseases ranging from chronic respiratory tract to urinary tract infections and blood infections [9]. This opportunistic bacterium was considered a significant threat by the Centers for Disease Control and Prevention (CDC) in a 2013 antibiotic resistance threat report [10] as a result of the increasing development of multidrug resistant (MDR) strains leading to a lot of therapeutic challenges due to the absence of effective treatment and consequently high rates of mortality [11,12]. The outer membrane permeability of *P. aeruginosa* is low, acting as an intrinsic barrier and making it resistant to several antibiotics [13]. When subjected to antibiotic pressure, this bacterium is able to adapt to this condition through an enhanced metabolic response that promotes the bacterial survival and antibiotic resistance development [14]. Because this bacterium has low nutritional requirements and adaptable energy metabolism, it can adjust to conditions that other organisms cannot tolerate [15]. Moreover, strains of *P. aeruginosa* can form a biofilm making their cells insensitive to disinfectants or host defense mechanisms [16]. Such pathogenesis results from the production of various virulence factors during infection, permitting it to survive and colonize its hosts [17]. Some of these factors are: type IV pili (the main adhesion to epithelial cells) [18], Exotoxin A (toxin that causes host cells death by necrosis) [19] and metallophores (secondary metabolites for metal ions sequestering) [20]. Like other bacteria, *P. aeruginosa* directly interacts with the extracellular medium in order to control and constantly maintain the intracellular metal ions concentration [21]. Metals are involved in their development and survival through a regulated network that includes several specific metal–DNA binding transcriptional regulators [22]. Most enzymes utilize metal ions as co-factors, and when the availability of one essential metal ion is low, some essential enzymes needed for metabolism cannot function properly, thus affecting cellular growth. On the other hand, excess metal ions can cause toxicity even if they are necessary for bacterial survival [23].

*P. aeruginosa* has powerful metal acquisition pathways enabling it to withstand growth conditions changes. They produce metallophores that chelate metal ions from the surrounding environment especially in minimum media. One of the strategies used by *P. aeruginosa* to uptake iron, which is a key element for this bacterium pathogenesis, is siderophores production. The latter are small ferric iron ions chelators that can scavenge and transport iron ions from the extracellular environment via specific protein receptors located on the outer membrane [24,25].

The first siderophore is pyoverdine; it has high iron chelating affinity, and it has been reported that strains unable to produce pyoverdines had reduced virulence during mice infections [26]. The importance of pyoverdines in the virulence of this bacterium was also proved using mouse and rabbit lung infection models [27,28]. It was found that pyoverdines play a dual role during infection. In addition to iron scavenging, they act as signaling molecules for the production of two vital virulence factors (the endo-proteinase PrpL and exotoxin A) [29,30]. The other siderophore produced by this bacterium is pyochelin, although its iron affinity is lower than that of pyoverdine [31] and fewer genes are involved in its biosynthesis [32]. It has been indicated that pyochelin is firstly produced by *P. aeruginosa,* but only when iron concentration becomes extremely low does this bacterium starts producing pyoverdine [33]. Pyochelin production might have a role in the continuous inflammatory response that cause tissues damage in chronic infections, such as in cystic fibrosis lungs [34]. *P. aeruginosa* also produces a third metallophore specific for the uptake of metal ions other than iron. It is a narrow spectrum metallophore called pseudopaline [35]. Pseudopaline is related to the nicotianamine of plants and it is a an opine-type metallophore that belongs to the same family of staphylopine synthetized by *Staphylococcus aureus (S. aureus)* [36]. This review highlights the three metallophores produced by *P. aeruginosa* (pyoverdine, pyochelin and pseudopaline), their synthesis pathways, and export and import systems in addition to their regulation at a genetic level and the important role they play in the virulence of this pathogen.

It worth mentioning that all the references used In this review were cited from trusted and impacted scientific platforms, mainly PubMed and MEDLINE. The reference papers were based on the previous research conducted on the three metallophores produced by *P. aeruginosa.*

## 2. Pyoverdine

Iron is crucial for bacterial cellular function, but at physiological pH (near neutral), iron has low solubility and is not available freely to pathogens. This low bioavailability leads to competitive interactions for iron between host and pathogen [37]. Pathogens have evolved sensitive systems for detecting low intracellular iron and stimulating siderophore production [38,39].

**Figure 1:**
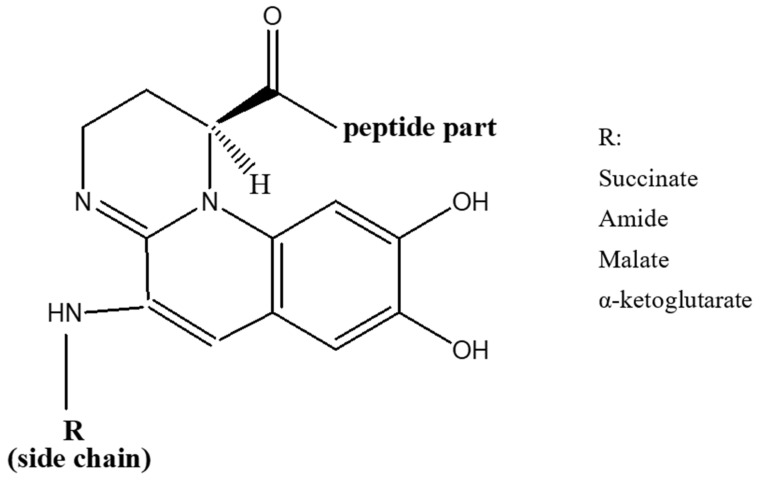
General structure of pyoverdines.

Pyoverdines were first discovered in 1892 [40], and their role in the acquisition of iron by *Pseudomonads* was indicated in late 1970s [41]. Currently, over 100 pyoverdines secreted by different *Pseudomonas* strains and species have been identified [42] which represent about 20% of the characterized microbial siderophores [43]. Their general chemical structure contains three bidentate chelating sites, including two hydroxamates and a catechol (Figure 1). These siderophores are made up of three parts: a peptide specific for each strain with a sequence of 6 to 12 amino acids linked to the carboxyl group, a chromophore part derived from 2,3-diamino-6,7-dihydroxyquinoline which is responsible for their fluorescence [44], and a side-chain linked at C-3 position to the chromophore nitrogen atom (NH_2_ group). In the majority of cases, the side-chain is a Krebs cycle diacid, such as malic, succinic or one of their amide derivatives [45].

The conserved chromophore part among all *Pseudomonas* species and strains chelates Fe^3+^ via the function catechol. The peptide moiety interacts with Fe^3+^ via two functions (hydroxamate and/or hydroxycarboxylate) and its sequence differs between each *Pseudomonads* species as well as strains of the same species [46]. Moreover, it can be linear or cyclic, and comprise unusual amino acids (e.g., D-isomers) and others that are not usually found in biomolecules [47].

Three structurally distinct types of pyoverdine are produced by *P. aeruginosa* strains (class I, II and III); each has its characteristic peptide chain [47]. The Pyoverdines produced by fluorescent *P. aeruginosa* strains are presented in Table 1.

Pyoverdines of class I (PVDI), produced by *P. aeruginosa* PAO1, are characterized by a cycle formed by the last four amino acids of the peptide moiety. An amide bond is formed between the carbonyl group of the *C*-terminal amino acid and the ε-amino group of an in-chain Lysine or Ornithine residue. On the other hand, the peptide sequence of class II pyoverdines (PVDII), produced by *P. aeruginosa* ATCC 27853, is linear with the *C*-terminal amino acid being of the *N*-hydroxy (cyclo)Ornithine type. As for class III pyoverdines (PVDIII), produced by *P. aeruginosa* Pa6, they have a linear peptide sequence with an unmodified *N*-hydroxyOrn at the *C*-terminus [47]. Iron acquisition mechanisms have been investigated the most in *P. aeruginosa* PAO1. A fourth pyoverdine that belongs to the strain R’ was also isolated where it was classified within the same siderovar (sv.) like strain Pa6 (sv. III). However, due to the variation in the structure of pyoverdine between the two strains, it was proposed to classify R’ strain in a sub-group (sv. III-2) of *P. aeruginosa* sv. III, different from the Pa6 sub-group (sv. III-l) [51].

### 2.1. Biosynthesis and Secretion of Pyoverdines

The biosynthesis of pyoverdines starts in the cytoplasm and terminates in the periplasm, to be secreted into the extracellular medium. Pyoverdines regulate their own production since they are quorum sensing molecules (important gene regulation mechanism in many bacteria), so a small amount of pyoverdine is constantly produced [52]. Several enzymes are included in the synthesis process, including in particular non-ribosomal peptide synthetases (NRPSs) [53]. These are large enzymes that contain multiple modules, where each module catalyzes the inclusion of a specific amino acid into the peptide product and forming peptide bonds between them [54]. Both the chromophore backbone and the peptide moiety are synthesized by NRPSs. The diversity of the distinct pyoverdines peptides sequences is a result of the different substrate NRPSs specificities in different strains and species [55]. The synthesis of pyoverdine is best understood and intensively investigated for the strain PAO1 of *P. aeruginosa* that produces PVDI.

The genes that are responsible for pyoverdine are situated on the *pvd* locus in the strain PAO1. The four largest genes, *pvdL*, *pvdI*, *pvdJ* and *pvdD* encode NRPSs [56]. Pyoverdines incorporate non-conventional moieties, such as chromophore, so NRPSs incorporate and link these moieties into the siderophore, which are supplied by the enzymes PvdA, PvdF and PvdH present in the cytoplasm [45]. Consequently, PVDI biosynthesis starts in the cytoplasm where a periplasmic peptide precursor forms designates as ferribactin (a yellowish non-fluorescent compound). Ferribactin is different from PVDI because it contains the tripeptide L-Glu–D-Tyr–L-Dab in place of the chromophore.

The first step in the PVDI synthesis starts with the enzyme PvdL that couples fatty acid (myristic or myristoleic) to a coenzyme A. PvdL is an atypical PVD-synthesis among NRPSs since it does not contain an initial C-terminal domain and includes an unusual domain which is related to acyl coenzyme A ligases [57]. The second step is the incorporation of the coenzyme A–fatty acid complex with an L-Glu moiety by PvdL. The main purpose of this fatty acid presence is to keep the precursor in the inner membrane [58]. The hydrolysis of fatty acid occurs prior to the siderophore excretion outside the cell. Then, PvdL integrates _D_-Tyr and _L_-Dab moieties that are condensed together to form a tetrahydropyrimidine ring that is the precursor to the dihydroxyquinoline chromophore [57]. In the final step, PvdL catalyzes the addition of the thus formed pyoverdine precursor to a _D_-Ser amino acid, which is the first amino acid of PVDI peptide moiety. The only NRPS present in all *Pseudomonas* genome is PvdL [57]. The PvdI and PvdJ enzymes further elongate the peptidic part through condensation and partial cyclization of eight amino acids. The enzyme PvdH catalyzes _L_-Dab synthesis, while PvdA and PvdF catalyze the formylhydroxyornithine synthesis [59,60]. In the end, the PvdD enzyme terminates the peptidic part via the activity of its thioester domain that enables ferribactin release into the cytoplasm. Subsequently, this molecule will be exported across the inner membrane by PvdE ABC-transporter [61].

**Figure 2:**
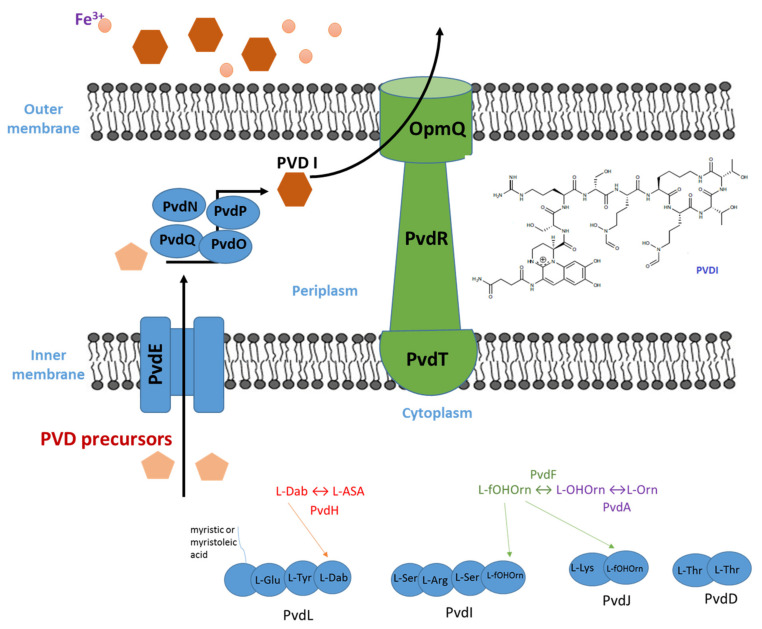
Biosynthesis of pyoverdine I.

The molecule is matured further in the periplasm to give the fluorescent form of pyoverdine. The enzymes PvdN, PvdO, PvdP and PvdQ are implied in the maturation biochemical events that lead to the formation of chromophore [62,63]. The PvdQ acylase enzyme detaches the fatty acid chain, giving ferribactin [64]. Pyoverdine production meets deacylation of the ferribactin precursor–quorum signaling. PvdQ was initially discovered as a QS-quenching enzyme that deacylated the autoinducer N-(3-oxododecanoyl)-L-homoserine lactone (3-Oxo-C12-HSL) [65]. The remaining three enzymes involved are probably required for the chromophore cyclization, although their specific functions are not fully characterized [61]. The chromophore formation is a multistep oxidative process [66], where ferribactin is transformed into dihydroPvd, a non-fluorescent and an unsaturated form of pyoverdine. DihydroPvd coexists with PVD in culture supernatants of *Pseudomonas* species [67]. Ferribactin is converted into PVDI by PvdP in three steps [68]. Firstly, _D_-Tyr is hydroxylated to a catechol moiety. Then, the third chromophore ring forms giving the final PVDI chromophore. Finally, the catechol moiety responsible for providing the third iron chelation site to pyoverdine is restored. It is presumed that PvdN and PvdO could be required in the side-chain conversion. After that, the PVD is released into the extracellular medium by the efflux pump PvdRT-OpmQ [69]. The latter have similar organization as other efflux pumps bacteria, where PvdT is the inner membrane protein, PvdR being a periplasmic adaptor protein and OpmQ is the outer membrane protein having a β-barrel domain (inserted in the outer membrane) and a large periplasmic extension [70]. The biosynthesis pathway is illustrated in Figure 2.

As mentioned before, PVDs differ only by the peptide sequence. The enzyme PvdL, which is implicated in the chromophore precursor synthesis, is the only conserved NRPS, contrary to the downstream NRPSs, incorporated in the synthesis of the peptide backbone, that do not show many similarities. Therefore, the pyoverdines diversity is a result of the specificities of the NRPSs found in several *Pseudomonas* strains [55]. Genomic studies of several *Pseudomonas* species and strains, showed that the *pvd* locus is the most divergent one thus causing pyoverdine diversity [71].

### 2.2. PVD-Fe Complex Uptake

After extracellular iron chelation, the bacterium will uptake the complex PVD-Fe^3+^ in order to internalize iron. This complex is recognized by FpvA which is a specific membrane protein transporter, for iron loaded pyoverdines. Each PVD class is identified by its specific FpvA transporter (i.e., FpvAI, FpvAII and FpvAIII). PVDI, secreted by *P. aeruginosa* strains, is realized by the outer membrane FpvAI transporter [72], the only known pyoverdine transporter whose structure has been well characterized [73] contrary to FpvAII and FpvAIII [74]. FpvA is encoded by the *fpvA* gene. Moreover, a second alternative transporter named FpvB has been identified in most *P. aeruginosa* strains for PVD-Fe^3+^ complexes, yet it is less effective than FpvA [75]. FpvA, as the other outer membrane siderophore transporter, is TonB dependent [76]. Bacterial TonB-dependent transporters are proteins found in the outer membrane that bind and transport siderophores.

FpvA is made up of four domains: (1) the β-barrel domain, (2) the plug, which is a round *N*-terminal domain involved in pyoverdine signaling and is highly mobile [77], (3) a periplasmic signal sequence, and (4) the TonB box, located on the plug *N*-terminal domain of the plug.

The binding pocket of pyoverdine is located at the FpvA extracellular side [78]. The chromophore interacts largely with the plug domain while the peptide part is linked to the β-barrel domain. Although the siderophore outer membrane transporters binding sites are selective for one siderophore only, it has been found that they are able to recognize and transport PVDs produced by other *Pseudomonas* strains [79]. Upon siderophore binding, the FpvA transporter undergoes conformational changes. The release of Fe^3+^ cation process is still not fully known, but it should involve a periplasmic reductive process without PVD degradation or chemical modification [80]. It has been indicated [81] that the FpvCDEF ABC-transporter could be involved in the dissociation iron. This transporter has two periplasmic binding proteins, FpvF and FpvC, which are associated with the FpvE ATPase and the FpvD permease that bind PVD-Fe^3+^ in the periplasm iron release. The inner membrane proteins FpvG and FpvH are essential for iron release, and there is indirect evidence that FpvG catalyzes the reduction step. Once released, FpvDE will transport it to the cytoplasm, and the *apo*-PVD (iron free) is recycled and transported back to the extracellular medium through the PvdRT-OpmQ efflux pump, in the same pathway of newly synthesized PVD [80]. The stability constant of the 1:1 Fe^3+^-pyoverdine complex was determined as K_a_ = 10^30.8^ M^−1^, and the corresponding value for the 1:1 Fe^2+^-pyoverdine complex as K_a_ = 10^9^ M^−1^ [82].

**Figure 3:**
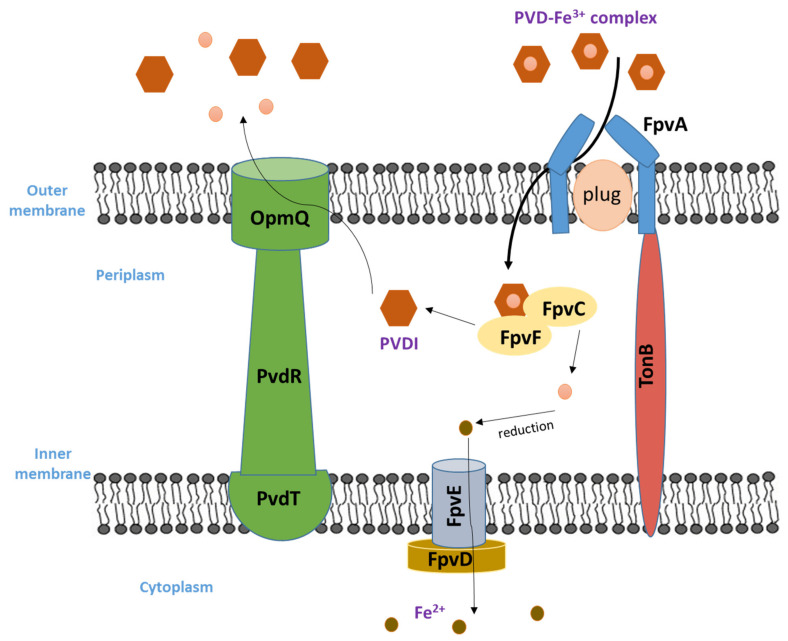
The pathway of iron loaded PVDs uptake and the excretion of recycled ones.

Figure 3 illustrates the uptake of iron loaded pyoverdine.

The PvdRT-OpmQ pump in *P. aeruginosa* is involved as well in PVDI–metal complexes efflux present in the bacterial periplasm other than PVDI–Fe^3+^ [83]. Siderophores can effectively chelate various metals other than iron [63] and this is the case for as well PVDI, which can also chelate Cd^2+^, Cu^2+^, Ni^2+^ and Zn^2+^ [84]. However, these PVD–metal complexes are not further transported into the cells of the bacterial, but are exported back directly from the periplasm into the extracellular medium via PvdRT-OpmQ. Currently, there is no available data that prove the dissociation of metal ions, other than Fe^3+^, from PVDI in the periplasm of *P. aeruginosa* [82].

### 2.3. Storage of PVDI in the Periplasm

It was found that the cells of *P. aeruginosa* store newly synthesized PVDI in their periplasm which enables the bacterium to stop undesired metals to the cytoplasm. If a toxic metal ion diffuses through porins into the periplasm, then PVDI, present in elevated concentration, will chelate the metal and the complex formed will be excreted by PvdRT-OpmQ. Thus, apart from iron, other metals have small possibility to enter into *P. aeruginosa* cells cytoplasm unless their affinity for a specific binding protein in the periplasmic is high. Consequently, having high stored PVDI concentration in the periplasm might help in avoiding the diffusion of unwanted metals into the cytoplasm, regulating the intracellular metal ions concentration and avoiding non-specific metal–protein interactions. Such PVDI biosynthesis cellular organization protects *P. aeruginosa* against iron disruption or other metal homeostasis, so the storage of PVDI in the periplasm indicates that siderophores might have other functions than sequestering iron [45].

### 2.4. Bacterial Virulence and PVDs

Besides being siderophores, pyoverdines act as signal molecules that trigger virulence factors production. PVDs compete for iron with the host iron-containing proteins such as transferrin [85]. Moreover, PVDs regulate the exotoxin A production (a lethal cytotoxin), an endoprotease PrpL (hydrolyze lactoferrin, transferrin etc.) and pyoverdine itself [29]. Signaling starts when Fe-PVDs interact with the transporter FpvA and is transduced via the FpvR anti-σ factor. The protein FpvR, which is located in the inner membrane, has parts in both the cytoplasm and the periplasm and chelates two extracytoplasmic σ factors PvdS and FpvI. The Fe–PVD binding onto FpvA induce conformational change causing FpvR proteolysis and liberation of the two σ factors. Then, the activation of PvdS (cytoplasmic regulatory protein) occurs where it interacts with RNA polymerase thus triggering pyoverdines, exotoxin A and PrpL protease production [86]. In addition, it is well established that iron has a role in the formation of biofilm of *P. aeruginosa* where the active transport of chelated iron was found to be the signal for biofilm development [87]. Eventually, PVDs can be taken over to serve several purposes such as “Trojan horses” in order to deliver antibiotics into the bacterium, leading to its death.

### 2.5. Iron Uptake Regulation

The conserved ferric uptake regulator, Fur, a protein found in various Gram-negative bacteria, acts as an iron uptake genes repressor (biosynthesis of siderophore, receptors) once it binds to its Fe^2*+*^ co-repressor [88]. Previous studies showed that various genes are regulated by iron in *P. aeruginosa* [89,90]. Fur can either directly or indirectly control some genes implicated in the uptake of iron through extra-cytoplasmic sigma factors (ECF σ) or other regulators. PvdS is one of these ECF σ; it controls pyoverdine biosynthesis genes transcription, along with the virulence genes like those encoding exotoxin A or PrpL (extracellular protease) [52]. In the *P. aeruginosa* PAO1 genome, there are 19 ECF sigma factors coding genes [91,92]. Some of these genes are Fe-regulated, including *pvdS* and *fpvI*, that code for ECF σ and are required for pyoverdine biosynthesis transcription genes and the TonB dependent Fe-pyoverdine receptor gene *fpvA*, respectively [93]. Fur indirectly regulates the expression of 11 TonB-dependent receptor genes through ECF σ factors while another 10 TonB-dependent receptors genes are directly Fur regulated [94].

## 3. Pyochelin

**Figure 4:**
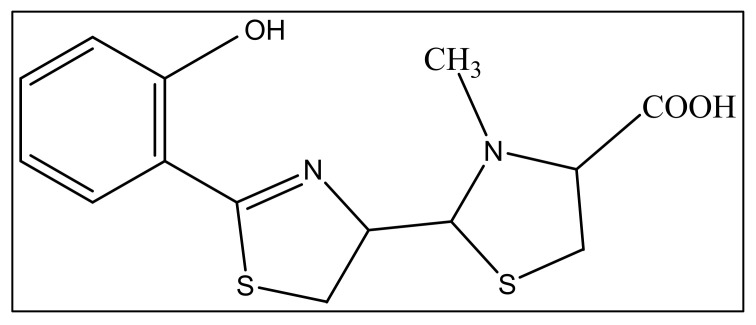
The structure of pyochelin.

The second siderophore that is produced by *P. aeruginosa* is pyochelin (PCH) with lower iron affinity than pyoverdine as previously mentioned. It is a member of phenols, a monocarboxylic acid and a member of thiazolidines produced by salicylic acid condensation with two cysteine molecules [95]. The pyochelin structure is shown in Figure 4.

### 3.1. Pyochelin Biosynthesis

Pyochelin is a nonribosomal peptide like pyoverdine and contributes to *P. aeruginosa* virulence as well [96,97]. Its biosynthesis requires NRPS which are large multidomain where each domain module conducts the addition of a single amino acid to the developing peptide chain. A module of NRPS consists of a C-domain (condensation), an A domain (adenylation) and a P domain (peptidyl carrier protein). The first initiation module is an exception since it lacks a condensation domain. The elongation of the chain is terminated by a T domain (thioesterase) in the terminal module [98,99]. Three precursor molecules form pyochelin: one salicylate molecule, two cysteine molecules and a hydroxy acid. The genes involved in pyochelin formation are organized in two operons, *pchDCBA* and *pchEFGHI* [100,101]. Three NRPS modules are required for the PCH biosynthesis: an initiation module that lacks the C-domain, an elongation one and a T-domain termination module. Pyochelin biosynthesis is initiated by two accessory enzymes, PchA and PchB [32,102]. PchA is an isochorismate synthase that converts chorismate to isochorismate, [103] and PchB is an isochorismate pyruvate lyase that subsequently converts isochorismate to salicylate [104]. Then the A-domain, PchD, activates the salicylate and transfers it to the NRPS *N*-terminal P-domain, PchE (the initiation module) [105]. The elongation module of PchE integrates L-cysteine into the emerging peptide chain, cyclizes the L-cysteine to a thiazoline ring and alters the stereochemistry with an epimerase tailoring domain [106]. The PchF termination module then adds and cyclizes a second L-cysteine in order to generate a second thiazoline. The latter is reduced to thiazolidine via the PchG tailoring protein [97]. The PchF methyltransferase domain performs an S-adenosylmethionine dependent thiazolidine N-methylation [103]. Finally, the PchF T-domain releases the mature pyochelin.

### 3.2. Pyochelin–Fe Uptake and Regulation

**Figure 5:**
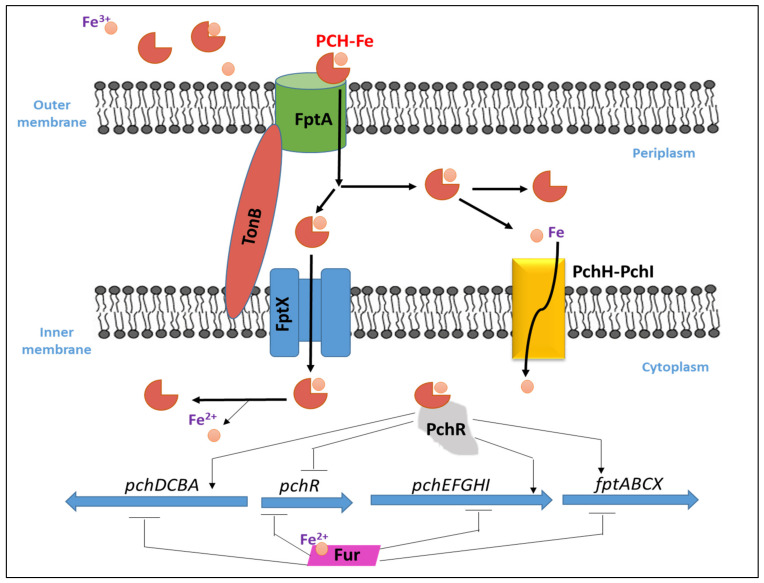
Iron uptake in *P. aeruginosa* and its transcriptional regulation with PCH.

In *P. aeruginosa*, once PCH chelates iron in the extra-cellular environment, the PCH–Fe complex is imported back into the periplasm through FptA [92] which is a specific outer membrane transporter (TonB dependent transporter) and encoded by the *fptA* gene, then into the cytoplasm via the permease FptX [107]. The PCH–Fe complex is required for its own because it binds and activates the transcription regulator PchR in the cytoplasm [97]. The transcription factor, PchR, belonging to the AraC-type transcription regulator family activates the PCH pathway. Upon the entry of PCH–Fe complex into the periplasm, a fraction of it is transported by FptX into the cytoplasm to interact with PchR as noted before, while a second PCH–Fe complex fraction will dissociate in the periplasm through an unknown mechanism, and the free iron ions will be further transported into the bacterial cytoplasm across the inner membrane via PchHI [108]. The two operons of the PCH biosynthesis genes (*pchDCBA* and *pchEFGHI*), are located next to the *pchR* regulatory gene and to the PCH–Fe transport operon *fptABCX*. Fur represses these three operons when iron is present and are activated by the AraC-type regulator PchR together with PCH when iron is scarce [109]. PCH binds Fe^3+^ with a 2:1 stoichiometry (Pch to Fe^3+^) and a stability constant of 2 × 10^5^ M^−1^ which is considered low for a siderophore [110]. The mechanism of iron uptake by PHC in *P. aeruginosa* and its transcriptional regulation is illustrated in Figure 5.

### 3.3. PCH Metal Chelation

In addition to iron sequestering, PCH can chelate Zn^2+^, Cu^2+^, Co^2+^, Mo^6+^ and Ni^2+^ [111], as well as non-biologically relevant cations like Ga^3+^ [112]. Thus, PCH has a role in uptake of biologically relevant metal ions other than Fe^3+^ even if its iron selectivity is very weak compared to other siderophores [31]. The uptake studies of several PCH–metal complexes showed that FptA has a large metal binding specificity. It can bind different PCH–metal complexes but PCH promotes the transport and accumulation only for Co^2+^ and Ni^2+^ in *P. aeruginosa* with uptake rates 23- to 35-fold lower than that for Fe^3+^ [110]. Thus, the role of PCH in cobalt and nickel uptake cannot be ignored. Although Zn^2+^, Cu^2+^, Co^2+^ and Ni^2+^, are enzymes cofactors with important roles in bacterial metabolism, they become toxic at high extracellular concentration [113]. Thus, free metal ions chelation in the extracellular medium can limit metals penetration by passive diffusion via the porines. These findings prove that PCH significantly increases the tolerance of *P. aeruginosa* to toxic metals, through the prevention of a lethal intra-bacterial metal overload [114]. It is worth mentioning that the FptA/PCH system does not intake large amounts of metals other than Fe^3+^. The binding pocket of PCH is principally composed of aromatic and hydrophobic residues (Phe114, Leu116, Leu117, Met271, Tyr334, Gln395 and Trp702), consistent with the hydrophobicity of a siderophore. PCH loaded with iron provides a tetradentate coordination. This siderophore has three chiral centers at positions C4′, C2′’ and C4′’. The removal of C4′ did not affect the iron binding properties or its uptake ability. On the other hand, with the removal of both chiral centers C4′ and C2′’, PCH still binds to FptA but was found to be unable to transport iron [110].

## 4. Uptake of Siderophores Produced by Other Microorganisms

Besides pyoverdine (PVD) and pyochelin (PCH), *P. aeruginosa* detects the presence of exosiderophores excreted by other bacteria in its extracellular medium via sigma and anti-sigma factors which is an AraC family transcriptional regulator [90]. These regulators are responsible for the transcription activation of exosiderophore-corresponding TBDT (TonB-dependent Transporters) in the presence of Fe–exosiderophore as well as the proteins needed for iron release from the chelator once it enters the bacteria [114,115]. The exosiderophores presence induces both the transcription and expression of their corresponding TBDTs in iron limited medium. A study was done in order to investigate how *P. aeruginosa* acts in the presence of several exosiderophores such as Enterobactin (ENT), an *E. coli* catechol siderophore [116] and vibriobactin (VIB), which is another catechol siderophore secreted by *Vibrio cholera* [117]. These exosiderophores were found to be able to induce the transcription and expression of specific TBDTs and repressed the transcription and expression of *fptA* (PCH specific TBDT), along with all genes related to PCH pathway. On the other hand, no effect on the PVD pathway expression genes was observed, as well as the ferrous and heme uptake pathways, or those for any other TBDTs [118]. These results indicated that the bacterium utilizes the triscatechol siderophores to sequester iron, instead of their own siderophores. This great phenotypic plasticity related to diverse pathways for up taking iron present in the genome of *P. aeruginosa,* gives it a high adaptation potential of in a variety of biotopes [119].

## 5. Pseudopaline

**Figure 6:**
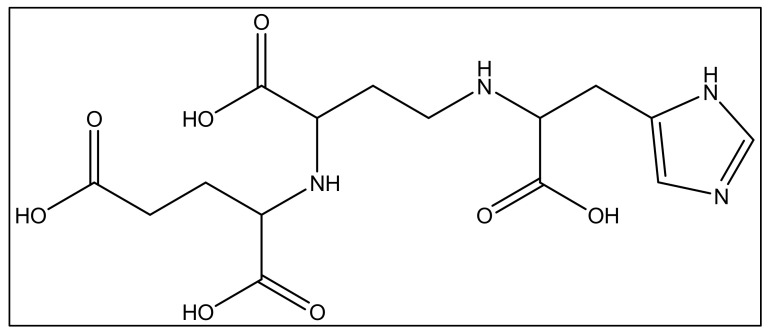
The structure of the opine-type metallophore in *P. aeruginosa* (pseudopaline).

In addition to iron, zinc is considered an essential metal ion required almost all living organisms at low concentrations [120], where it comes after iron in abundance [121]. In the opportunistic bacteria *P. aeruginosa*, it is well known that zinc plays an essential role in its virulence, host organism colonization, and antibiotic resistance [120]. Also, this bacteria needs zinc to resist carbapenems [122] as well as the extracellular proteases activity [123]. *P. aeruginosa* has several transport systems for zinc that enable it to survive in zinc scarce environments upon infection. In addition to PVD and pyochelin PCH, *P. aeruginosa* produces another metallophore called pseudopaline, biosynthesized by two enzymes, CntL and CntM [124]. Pseudopaline is plant nicotianamine related belonging to the same opine-type metallophore family as staphylopine and yersinopine synthetized by *S. aureus* and *Y. pestis,* respectively [36,125]. Various studies proved that pseudopaline contributes to the virulence of *P. aeruginosa*. The receptor CntA of pseudopaline is related to lung infections in cystic fibrosis patients, and the pseudopaline operon was highly upregulated in the infected host sites [126,127]. Besides zinc, pseudopaline is also involved in the uptake of cobalt [122] and nickel [35]. Urease, which is a nickel-dependent enzyme, is produced by *P. aeruginosa* [128]. Moreover, *P. aeruginosa* requires cobalt for the cobalamin-dependent ribonucleotide reductase (NrdJab) that is essential for developing biofilm under oxygen-limited conditions [129]. These findings demonstrate the importance of cobalt and nickel uptake in *P. aeruginosa* pathogenesis and the potential role of pseudopaline in sequestering these trace metal ions. The structure of pseudopaline is illustrated in Figure 6.

### Pseudopaline Biosynthesis, Excretion, Import and Regulation

**Figure 7:**
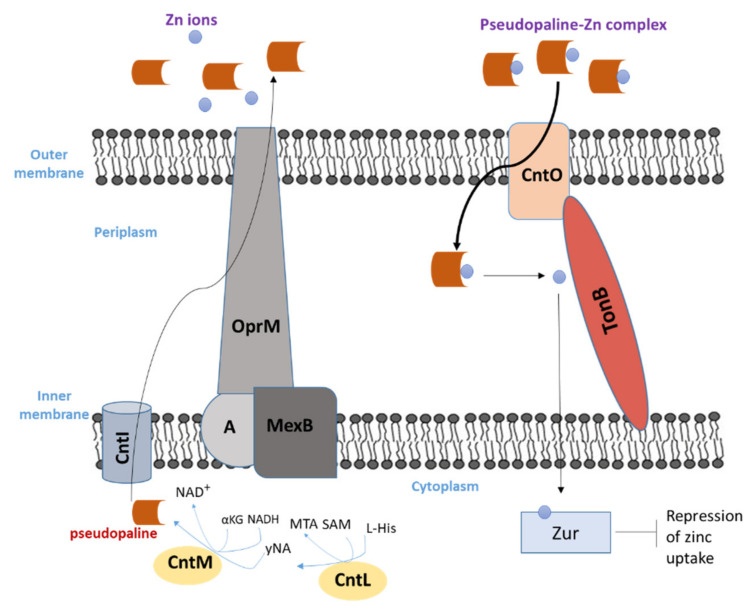
Pseudopaline biosynthesis, export, import and regulation process by *P. aeruginosa*.

The *P. aeruginosa cntOLMI* genes encode the proteins responsible for the biosynthesis and transport of pseudopaline [129]. They are all clustered together in a single operon regulated negatively by zinc ions via the Zur repressor [123]. *cntO* is the first operon gene; it encodes for the outer membrane component CntO which belongs to the TonB-Dependent Transporter (TBDT) family involved in the recovery of pseudopaline from the external medium. The second gene (*cntL*) and third gene (*cntM*) of the operon encode the two cytoplasmic enzymes CntL and CntM, respectively, which are responsible for the two-step biosynthesis process of pseudopaline. In the first step, yNA (a reaction intermediate) is produced by the enzyme CntL which uses S-adenosine methionine (SAM) and L-histidine as substrates. The second step is the NADH reductive condensation of yNA with a α-ketoglutarate (αKG) molecule, catalyzed by CntM in order to produce pseudopaline [123,124,125,126,127,128]. yNA presents a chemical resemblance with xNA (in staphylopine biosynthesis) and nicotianamine (NA). *cntI,* the fourth gene of the *cnt* operon, encodes CntI, an inner membrane protein, that belongs to the EamA or drug/metabolite transporter (DMT) family responsible for the export of pseudopaline from the cytoplasm to the periplasm after its biosynthesis [129]. After the transport of pseudopaline to the cytoplasm via CntI, it is exported through the outer membrane via the efflux pump MexAB–OprM (Figure 7), so *P. aeruginosa* secretes pseudopaline through a two-step secretion system [129].

## 6. Conclusions

The bacterium *Pseudomonas aeruginosa* is a dangerous and opportunistic bacteria responsible for severe nosocomial infections, and chronic infections in cystic fibrosis patients. The virulence of this bacterium is associated with various factors such as metallophores and toxins that have an important role in the bacterial colonization, survival and tissues invasion. Metallophores are secondary metabolites secreted by bacteria and enables them to sequester metal ions necessary for their metabolism thus allowing the survival in metal scarce conditions. *P. aeruginosa* secretes three metallophores, pyoverdine, pyochelin and pseudopaline. Pyoverdines are siderophores synthesized under low iron conditions in order to sequester iron and cause infections. These molecules are composed of three parts, the chromophore part which is conserved among all *Pseudomonas* strains, a side-chain part linked to the chromophore and a peptide part that can be either linear or cyclic and is specific to each pyoverdine. In addition, *Pseudomonas* strains are capable of using xenopyoverdines produced by other bacteria giving them a competitive advantage and allowing them to save energy since synthesizing and excreting pyoverdines is an energy consuming process. *P. aeruginosa* can secrete another siderophore called pyochelin, but in lower amounts and presents much lower iron affinity than pyoverdine. Moreover, the structure of pyochelin is simpler, smaller, and it reaches the bacterium cytoplasm. On the other hand, pyochelin is firstly synthesized by *P. aeruginosa* then the bacterium switches to producing pyoverdines under lower iron concentration. Pseudopaline is the third metallophore produced by *P. aeruginosa;* it is an opine carboxylate narrow spectrum metallophore that can sequester zinc, nickel and cobalt.

## Figures and Tables

**Table 1:** Pyoverdines that are synthesized by *P. aeruginosa* strains.

Species	Strains	Pyoverdines Type	Peptide Sequences	Ref
*P. aeruginosa*	ATCC15692(PAO1)	PVDI	Ser–Arg–Ser–FoOHOrn–[Lys–FoOHOrn–Thr–Thr]	[48]
*P. aeruginosa*	ATCC27853	PVDII	Ser–FoOHOrn–Orn–Gly–Thr–Ser–cOHOrn	[49]
*P. aeruginosa*	Pa6 (R) (sv. III-l)	PVDIII-1	Ser–Dab–FoOHOrn–Gln–Gln–FoOHOrn–Gly	[50]
*P. aeruginosa*	R’ (sv. III-2)	PVDIII-2	Ser–Dab–FoOHOrn–Gln–FoOHOrn–Gly	[51]

Dab: 2,4-diaminobutyrate; Orn: ornithine; OHOrn: *N*-hydroxyornithine; FoHOrn: *N*-formyl-*N*-hydroxyornithine; [ ] are used to show cyclic peptides.

## Data Availability

No data was reported in this study.
